# The role of candidate pharmacogenetic variants in determining valproic acid efficacy, toxicity and concentrations in patients with epilepsy

**DOI:** 10.3389/fphar.2024.1483723

**Published:** 2024-10-30

**Authors:** Hady Yazbeck, Joe Youssef, Wassim Nasreddine, Abdullah El Kurdi, Nathalie Zgheib, Ahmad Beydoun

**Affiliations:** ^1^ Faculty of Medicine, American University of Beirut, Beirut, Lebanon; ^2^ Department of Neurology, Faculty of Medicine, American University of Beirut, Beirut, Lebanon; ^3^ Department of Biochemistry and Molecular Genetics, Faculty of Medicine, American University of Beirut, Beirut, Lebanon; ^4^ Department of Pharmacology and Toxicology, Faculty of Medicine, American University of Beirut, Beirut, Lebanon

**Keywords:** pharmacogenetics, VPA, epilepsy, efficacy, toxicity, concentrations

## Abstract

**Background:**

Antiseizure medications (ASM) exhibit considerable interindividual variability in terms of efficacy and adverse events. Genetic variation is thought to contribute to these differences in clinical outcomes. Specifically, the response to valproic acid (VPA), a widely used ASM, is influenced by multiple pharmacogenetic factors. However, and in contrast to other ASMs such as phenytoin and carbamazepine, there is a paucity of data on the association between VPA and various gene variants. The aim of this study was hence to evaluate the influence of candidate pharmacogenetic variants on VPA efficacy, toxicity and serum concentrations in a homogeneous cohort of patients newly diagnosed with genetic generalized epilepsies (GGE).

**Methods:**

In this prospective cohort study, demographic, clinical and treatment outcomes of GGE patients were retrieved from their medical records. Whole exome sequencing was performed in collaboration with Epi25. Gene variants associated with VPA efficacy, metabolism and toxicities were retrieved from PharmGKB. An analysis was then conducted to explore potential associations between these gene variants and VPA clinical outcomes.

**Results:**

Of the 166 patients included, 60 (36.1%) experienced treatment failure while 106 (63.9%) achieved treatment success. After adjusting for VPA maintenance dose, carriers of the rs3892097 (*CYP2D6*) variant were 2.5 times more likely to experience treatment failure compared to wildtype (*p* = 0.026). The rs1057910 variant (CYP2C9*3) was associated with increased serum VPA concentrations (*p* = 0.034). Moreover, the rs1137101 variant (*LEPR gene*, a metabolism regulator) was significantly associated with a higher risk of weight gain (regression coefficient of 3.430 [0.674; 6.186], *p* = 0.015) and a higher frequency of hair loss (OR = 3.394 [1.157; 9.956], *p* = 0.026), while the rs4480 variant (SOD2 gene, encoding for a mitochondrial scavenging enzyme) was correlated with a lower frequency of hair loss (OR = 0.276 [0.089; 0.858], *p* = 0.026).

**Conclusion:**

These findings highlight the role of genetic factors in VPA treatment and underscore the potential for developing therapeutic strategies to enhance patient outcomes and minimize adverse effects.

## 1 Introduction

Epilepsy, one of the most common neurological disorders, is characterized by recurrent, unprovoked seizures (Stafstrom and Carmant, 2015). Although its incidence is highest in infants and the elderly, epilepsy can manifest at any age, with diverse etiologies and clinical manifestations (Duncan et al., 2006).

Antiseizure medications (ASMs) control seizures in approximately 70% of newly diagnosed patients, thereby reducing morbidity and improving quality of life (Stafstrom and Carmant, 2015; Duncan et al., 2006). However, there is substantial interindividual variability in therapeutic response, dose-related adverse drug effects (ADEs), and drug plasma concentrations, even among patients with identical seizure types and treatment regimens (Loscher et al., 2009). Emerging evidence indicates that genetic variability substantially contributes to these differences in clinical outcomes (Loscher et al., 2009). This highlights the importance of pharmacogenetics, the study of genetic influences on drug response, in optimizing epilepsy treatment by elucidating the genetic factors influencing individual responses to ASMs.

Valproic acid (VPA), known for its broad spectrum of efficacy, is a widely used ASM, particularly in the management of genetic generalized epilepsies (GGE) (Marson et al., 2007; Marson et al., 2021; Epi25 collaborative, 2024). The pharmacogenetic landscape implicates numerous candidate genes in shaping inter-individual variability in VPA response based on its mechanisms of action and pharmacokinetic profile (PharmGKB, 2024c). For example, VPA enhances GABAergic activity by inhibiting ABAT and ALDH5A, enzymes crucial in gamma-aminobutyric acid (GABA) degradation (PharmGKB, 2024b; Johannessen and Johannessen, 2003). Additionally, VPA’s pharmacodynamic activity includes blocking voltage-gated sodium, potassium and calcium channels, including CACNA1C, CACNA1D, CACNA1N, and CACNA1F and the SCN family, thereby reducing high-frequency neuronal firing (Johannessen and Johannessen, 2003; Van den Berg et al., 1993). VPA undergoes extensive hepatic metabolism via enzymes such as UGT1A3, UGT1A4, UGT1A6, UGT1A8, UGT1A9, UGT1A10, UGT2B7 and UGT2B15, transforming VPA to valproate-glucuronide, its major urinary metabolite (Ito et al., 1990; Argikar and Remmel, 2009; Tan et al., 2010). Moreover, variants of the *ABCB1* and *ABCC1* gene encode efflux transporters at the blood-brain barrier that can expel VPA from the brain, lowering its concentration and potentially leading to VPA-resistant epilepsy (Kwan et al., 2009; Gibbs et al., 2004).

Despite its clinical significance, the pharmacogenetic data on VPA is limited compared to other ASMs such as carbamazepine and phenytoin, and the existing evidence is less robust (Ghodke-Puranik et al., 2013; Balestrini and Sisodiya, 2018). For instance, a multi-center study involving 142 adult Caucasian patients with epilepsy evaluated the association between the *T1405* polymorphism in the carbamoyl phosphate synthetase 1 (*CPS1*) gene and VPA-induced hyperammonemia, demonstrating an increased risk in patients with the *T1405* variant (Janicki et al., 2013). Similarly, a study on 52 pediatric patients with epilepsy showed that tailoring treatment according to *CYP2C9* status reduced the incidence of hyperammonemia (Bűdi et al., 2015). In another study, several single nucleotide polymorphisms (SNPs) in the *ABCB1* gene were analyzed in 170 Chinese children, revealing that homozygous carriers of rs1128503 were at higher risk of persistent seizures, while homozygous carriers of rs3789243 had a lower risk of gastrointestinal ADE (Zhu et al., 2023). However, the association between *ABCB1* polymorphisms and ASM resistance remains inconclusive, and routine testing to predict response is not currently recommended (Balestrini and Sisodiya, 2018). Furthermore, a cohort study involving 212 Han Chinese patients with epilepsy treated with VPA identified associations between weight gain and polymorphisms in the leptin receptor (*LEPR*) and ankyrin repeat kinase domain containing 1 (*ANKK1*) genes (Mould and Upton, 2013). However, all of these studies require further validation because the evidence remains ambiguous (Balestrini and Sisodiya, 2018).

To address current gaps in the literature, we conducted a prospective cohort study aiming to evaluate the influence of candidate pharmacogenetic variants on VPA efficacy, toxicity and serum concentrations in a homogeneous cohort of patients newly diagnosed with GGE.

## 2 Methods

The study was approved by the American University of Beirut Medical Center (AUBMC) Institutional Review Board (IRB) (IM.AB1.01). All subjects or parents signed a genetic informed consent, and children signed an assent form as applicable.

### 2.1 Prospective enrollment of patients with newly diagnosed epilepsy

The patients included in this nested pharmacogenetic study were recruited from an ongoing prospective study, initiated in 2010 and focusing on children and adults newly diagnosed with epilepsy. As of August 2024, a total of 3010 subjects have been enrolled (Jomaa et al., 2023). This multicenter study is centralized at the AUBMC and conducted in association with the Lebanese Chapter of the International League against Epilepsy (ILAE). Adult and pediatric neurologists from across Lebanon refer patients with suspected newly diagnosed seizures to the AUBMC where a comprehensive evaluation is conducted. The work-up on each patient includes a 3-h sleep deprived video-EEG recording interpreted by two experienced electroencephalographers along with an epilepsy protocol brain MRI interpreted by a neuroradiologist blinded to the patient’s history. The results of the work-up are communicated to the referring physician who decides whether to initiate treatment and selects the appropriate ASM. If treatment with an ASM is initiated, the dose is adjusted until achieving seizure freedom or the occurrence of intolerable adverse events. Patients are subsequently evaluated every 3 months during the first year, and every 6 months thereafter, with additional visits as needed based on seizure recurrence or adverse events. Patients who experienced absence, myoclonic or primary generalized tonic-clonic seizures with evidence of generalized spike wave discharges on EEG were diagnosed with a GGE (Hirsch et al., 2022).

### 2.2 Whole exome sequencing in collaboration with the Epi25 collaborative

In 2017, the ongoing AUBMC cohort joined the Epi25 Collaboration (Epi25) consortium, which includes collaborators from all over the world. The consortium aims to perform Whole Exome Sequencing (WES) on a large population of patients with epilepsy to elucidate the genetic influences on both rare and common forms of epilepsy and to investigate whether genetic risk originates from *de novo* mutations or inherited variants (Epi25 collaborative, 2024). The samples are sequenced at the Broad Institute using the Illumina Infinium Global Screening Array, GSA-MD v1.0 (Illumina, San Diego, United States) (Epi25 Collaborative. Electronic, 2021; Niestroj et al., 2020; Montanucci et al., 2023).

Peripheral blood was collected into EDTA tubes, aliquoted and stored at −80°C until DNA isolation using the Flexigene DNA isolation kit (Qiagen, Ca, United States) as per manufacturer’s guidelines. The isolated DNA was stored at −20°C.

By 2020, a total of 1238 peripheral DNA samples were shipped from AUBMC to the Board Institute for WES. Following sequencing, BAM and VCF files were returned for storage and analysis by the institutional Bioinformatics core lab. Raw reads in unaligned BAM format (uBAM) were aligned to the human reference genome build 38 (hg38) using Burrows-Wheeler Aligner (BWA) (Li and Durbin, 2010). The aligned reads were then sorted by chromosome coordinates, and duplicate reads were marked using the Genome Analysis Toolkit (GATK) MarkDuplicates tool (Van der Auwera et al., 2013). Base quality score recalibration was applied using the GATK ApplyBQSR tool. Single nucleotide variant (SNVs) and insertions/deletions (INDELs) were identified using the GATK HaplotypeCaller. The GATK GenomicsDBImport tool was used to merge GVCFs from multiple samples, followed by joint genotyping using the GenotypeGVCFs tool from GATK. Variant filtration and variant calling quality score recalibrations were performed using GATK VariantFiltration and Variant Quality Score Recalibration (applyVQSR) tools, respectively. The final variant call VCF file (format v4.2) was generated by the Broad variant calling pipeline with GATK version 4.1.1.0. Variants were annotated using Ensembl Variant Effect Predictor version 104 (Ensembl-VEP) (McLaren et al., 2016). Variants with a “PASS” filter tag were selected for downstream analysis. Variants and genotypes data extraction from the VCF file was performed using BCFtools (Danecek et al., 2021). The frequencies of the genetic polymorphisms were checked for conformity to the Hardy Weinberg Equilibrium (HWE) using the Chi-square test.

### 2.3 Nested sample for the current study

Patients included in this study had their samples sequenced up until 2020, were diagnosed with a GGE, initiated on VPA monotherapy, and had available clinical data. The following variables were collected from the patients’ charts, starting from the first visit to the last follow-up: gender, weight (kg), height (cm), age at diagnosis, electroclinical syndrome (ECS), VPA maintenance dose (mg/kg), VPA serum concentrations (mg/L) and corresponding doses, compliance, length of follow-up, and treatment discontinuation. Trough total VPA concentrations were determined from analysis of blood samples collected either eight to 12 h after the last VPA dose, or prior to the first dose of the day. Serum VPA concentrations were analyzed by a chemiluminescent micro-particle immunoassay (CMIA) using Architect from Abbott Laboratories (Abbott Park, Illinois, United States). The minimum detectable assay limit of the apparatus was 2 mg/L. At each follow-up visit or phone call, adherence to treatment was monitored through inquiries made to the caregiver/patient regarding the administration of ASM as prescribed. In addition, the duration of seizure remission was measured to evaluate treatment efficacy. Patients who were non-compliant and those with incomplete follow-up data were excluded from the study.

### 2.4 Selection of genetic variants

To identify target variants with genotype-phenotype associations involving VPA, a systematic search was conducted on 14 December 2023 using the “clinical annotations” and “variant annotations” feature of the PharmGKB curated database (PharmGKB, 2024a). This search focused exclusively on statistically significant (*p*-value <0.05) single nucleotide polymorphisms (SNPs), while excluding SNPs with unsupported evidence (level of evidence = 4). This search identified 31 variants of interest in 25 genes across four main phenotype categories: dosage, metabolism/pharmacokinetics, efficacy and toxicity. Further details on these variants, including classification based on VPA pharmacogenetic associations, mutations types and nucleotides changes, Genome Aggregation Database (gnomAD) (Genome Aggregation, 2024), and allele frequencies for all populations compiled and Europeans were extracted from the dbSNP database (Home - SNP - NCBI, 2024) (Supplementary Table S1).

### 2.5 Statistical analysis

Data were entered into SPSS (Version 25.0. Armonk, NY: IBM Corp.) to evaluate the potential associations between the various genotypes and treatment success, toxicity, and adjusted drug concentration of VPA for dose and weight (ADC) (Daci et al., 2015). Data are presented as mean ± standard deviation (SD) for continuous variables, and as counts and frequencies for categorical variables.

Patients who achieved at least 1 year of seizure freedom while on a maintenance dose of VPA monotherapy were categorized as having a successful treatment outcome. Conversely, patients who failed to achieve a 1-year remission, required treatment with an add-on ASM, switched medications after initiating VPA monotherapy, or discontinued VPA due to intolerable ADEs, were classified as treatment failures. Considered drug related toxicities included weight gain (calculated as the difference between the patient’s weight prior to VPA monotherapy from their last recorded weight while on VPA monotherapy), hair loss, nausea and/or vomiting and tremor.

For ADC analysis, all serum VPA concentrations were standardized by accounting for both the VPA dose and the patient weight using the following formula: serum VPA concentration (ug/mL)/[(patient weight (kg) * VPA dose (mg/day)], which yields the ADC variable in [(ug/mL/day)/(mg/kg)]. Weight values were recorded either at the time of VPA concentration measurement or within 6 months. If such data were unavailable, the average of the two closest weight measurements before and after the VPA concentration measurement was used. For patients with multiple VPA concentration readings at various time-points, the average ADC was computed.

Initially, a univariate association analysis was performed to assess the correlation of demographic variables and genetic polymorphisms with treatment success, toxicity, and ADC. Categorical variables were analyzed using the chi-square test. Continuous variables such as age at first visit, length of follow up, maintenance dose, VPA concentrations, and weight gain were assessed with the Mann–Whitney U test, after none were found to be normally distributed with the Shapiro-Wilk test for normality. The rest of the continuous variables were analyzed with Student t-test. Subsequently, a multivariate analysis was conducted to adjust for confounding variables. A binary logistic regression with Odds Ratios (OR) and 95% Confidence Intervals (CI) was performed to evaluate for the association with treatment success and toxicity (both categorized as yes/no), while a linear regression reporting Beta (β) and 95% CI was performed for ADC and weight gain. Confounding variables considered included gender, age at diagnosis, follow-up period and VPA dose per kilogram per day (mg/kg/d). Statistically significant (*p* < 0.05) confounding variables at the univariate level were adjusted for in the multivariate analysis. Significant differences in VPA maintenance doses were adjusted for in the treatment success and hair loss analyses. Significant differences in gender distribution were adjusted for in the nausea/vomiting and hair loss association analyses. Significant differences in age at initial diagnosis were adjusted for in the tremor and adjusted VPA concentration analyses. To account for children growth, we adjusted for age at first visit and length of follow-up in the multivariate analysis of the association of the gene variants with weight gain, even though age at first visit was not statically significant at the univariate level. Age at diagnosis was recorded as the age of the patient at the first visit. For VPA dose, the maintenance dose was used for cases of treatment success, the maximum dose for cases of inefficacy, and the dose at withdrawal for drug intolerability. The recessive genetic model was employed to evaluate the relationship between genes and outcome variables, comparing wildtype homozygous genotypes to the combined allele carriers and homozygous mutants.

## 3 Results

### 3.1 Final sample

Out of the 1238 sequenced samples, clinical data were reviewed for 218 consecutive patients who met the inclusion criteria. Fifty-two patients were excluded due to poor data quality, incomplete follow up, or non-compliance with medication. Thus, the final sample for the association analysis with treatment efficacy comprised 166 patients with an age range of 6 months–40 years (mean = 12.85 ± 7.28), and an initial weight range from 7.5 to 116 kg (mean = 47.32 ± 22.26, (N = 163)). For the toxicity analysis, four patients were excluded from the categories of hair loss (Supplementary Table S6) and nausea/vomiting (Supplementary Table S3) due to inadequate reporting of symptoms, thereby leaving 162 patients (N = 162) for the corresponding analyses. Three patients were excluded from the tremor analysis (Supplementary Table S4) for the same reason, thereby leaving 163 patients (N = 163) for the corresponding analysis. Weight gain analysis (Supplementary Table S5) was performed on 163 out of the initial 166 participants (N = 163), for which weight measurements were available. Finally, for the ADC analysis, VPA concentrations were available for only 150 out of the initial 166 participants (N = 150), on whom the corresponding analysis was conducted.

### 3.2 Genotyping results

Out of the 31 listed genetic variants, three were not detected as all patients were wildtype homozygous, one was excluded due to the absence of wildtype alleles in our population sample, six lacked sufficient depth of coverage, and four did not satisfy HWE. This resulted in a total of 17 genetic variants for the association analyses, with minor allele frequencies comparable to those observed in Europeans populations. Five variants (*rs2279020, rs9332120, rs3892097, rs7438284,* rs2269577) from five genes (*GABRA1, CYP2C9, CYP2D6, UGT2B7, XBP1)* were analyzed for treatment outcome (success versus failure), four variants (rs1137101, rs1800497, rs4880, rs3087374) from four genes *(LEPR, ANKK1, SOD2, POLG)* were assessed for toxicity, and eight variants (rs6759892, rs1105879, rs1105880, rs7592281, rs2070959, rs1057910, rs1799853, rs7668258) from three genes (UGT1A, *CYP2C9, UGT2B7)* were examined for adjusted VPA drug concentration based on the phenotype categories from the PharmGKB (Supplementary Table S1).

### 3.3 Association with VPA efficacy

Among the 166 patients included in the study, 60 (36.1%) experienced treatment failure while 106 (63.9%) achieved treatment success (Table 1). The treatment failure group had a mean maintenance VPA dose of 19.01 ± 9.03 mg/kg/d, significantly higher than the mean dose of 15.88 ± 6.7 mg/kg/d in the treatment success group (*p* = 0.036). This difference is expected, as patients resistant to treatment are likely to be prescribed higher doses of VPA. Other potential confounding factors did not show significant associations with treatment outcome.

**TABLE 1:** Association of demographics and genes variants with treatment efficacy at 12 months.

Variable name	All patients (N = 166)	No success of treatment at 12 months (N = 60)	Success of treatment at 12 months (N = 106)	*p*-value
Age at visit 1 (years)	12.85 ± 7.28	13.15 ± 6.65	12.68 ± 7.64	0.443
Maintenance dose per kg per day (mg/kg/d)	17.01 ± 7.75	19.01 ± 9.03	15.88 ± 6.7	**0.036**
Follow-up period (years)	6.58 ± 2.61	6.90 ± 2.66	6.40 ± 2.58	0.245
Gender (Female)	76 (45.78)	31 (51.67)	45 (42.45)	0.252
Gender (Male)	90 (54.22)	29 (48.33)	61 (57.55)	—
*rs2279020* (Wildtype)	36 (21.69)	16 (26.67)	20 (18.87)	0.241
*rs2279020* (Allele carrier + Homozygous mutant)	130 (78.31)	44 (73.33)	86 (81.13)	—
*rs9332120* (Wildtype)	107 (64.46)	34 (56.67)	73 (68.87)	0.115
*rs9332120* (Allele carrier + Homozygous mutant)	59 (35.54)	26 (43.33)	33 (31.13)	—
*rs3892097* (Wildtype)	135 (81.33)	44 (73.33)	91 (85.85)	**0.047**
*rs3892097* (Allele carrier + Homozygous mutant)	31 (18.67)	16 (26.67)	15 (14.15)	—
*rs7438284* (Wildtype)	55 (33.13)	18 (30.00)	37 (34.91)	0.519
*rs7438284* (Allele carrier + Homozygous mutant)	111 (66.87)	42 (70.00)	69 (65.09)	—
*rs2269577* (Wildtype)	69 (41.57)	22 (36.67)	47 (44.34)	0.335
*rs2269577* (Allele carrier + Homozygous mutant)	97 (58.43)	38 (63.33)	59 (55.66)	—

Mann-whitney U test was used for continuous variables and Chi-square for categorical ones. Continuous variables were reported as mean ± SD., Categorical variables were reported as N (%).

P-values in bold indicate statistically significant findings (P < 0.05).

**FIGURE 1:**
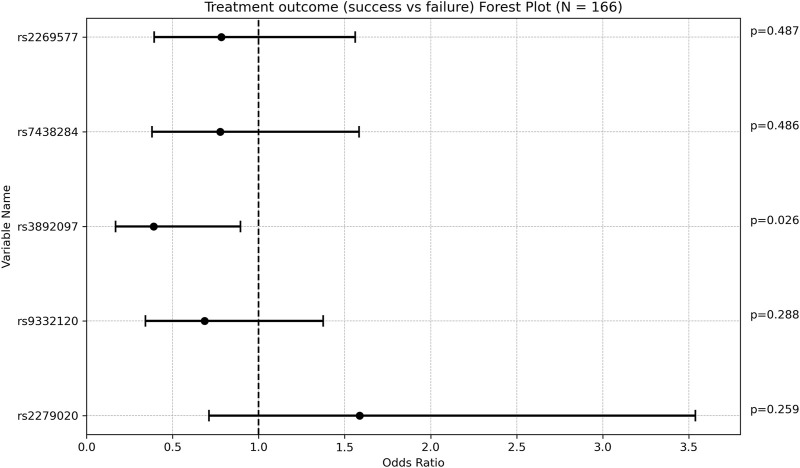
Forest plot representing the multivariate odds ratios (95%CI) of treatment success relative to treatment failure in each SNP.

Carriers of *rs3892097 (CYP2D6)* constituted 26.67% of the treatment failure group compared to 14.15% of the treatment success group (*p* = 0.047). The multivariate OR adjusted for maintenance VPA dose was 0.389 [0.169; 0.894] (*p* = 0.026), indicating that patients experiencing treatment failure were approximately 2.5 times more likely to carry this polymorphism. This underscores the detrimental effect of *rs3892097 (CYP2D6)* on the success rate of VPA monotherapy. No other SNPs showed significant associations with treatment outcome (Figure 1; Supplementary Table S2).

### 3.4 Association with VPA toxicity

In our study sample, ADEs from VPA monotherapy were spontaneously reported as follows: 4.93% of patients experienced nausea and/or vomiting, 12.26% had tremor and 11.72% reported hair loss.

All patients who experienced nausea and vomiting were females, whereas 42.86% of those without these symptoms were females (*p* = 0.002%). After adjusting for confounding variables, no SNP was found to be significantly associated with nausea and/or vomiting (Supplementary Table S3).

No statistically significant associations were identified between tremor and the genetic variants under investigation (Supplementary Table S4).

**FIGURE 2:**
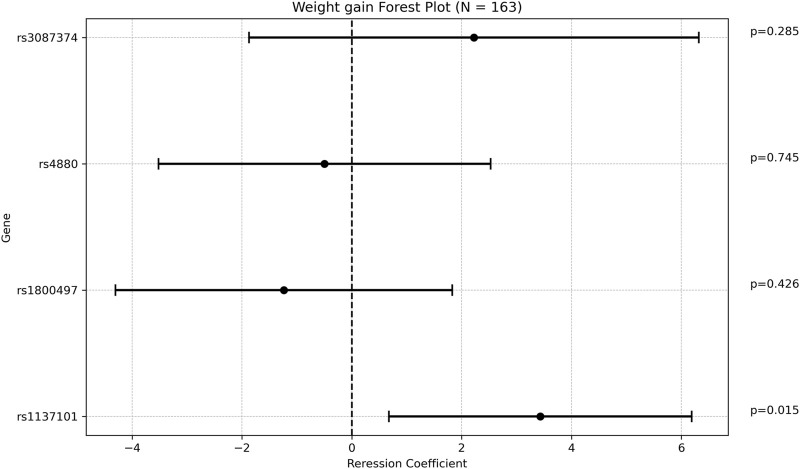
Forest plot representing the multivariate regression coefficients (95%CI) for weight gain in each SNP.

One SNP was found to be associated with increased weight gain after adjusting for VPA maintenance dose, age at diagnosis, and length of follow up. Specifically, *rs1137101 (LEPR)* had a multivariate regression coefficient of 3.430 [0.674; 6.186] with a *p*-value of 0.015. (Figure 2; Supplementary Table S5).

**FIGURE 3:**
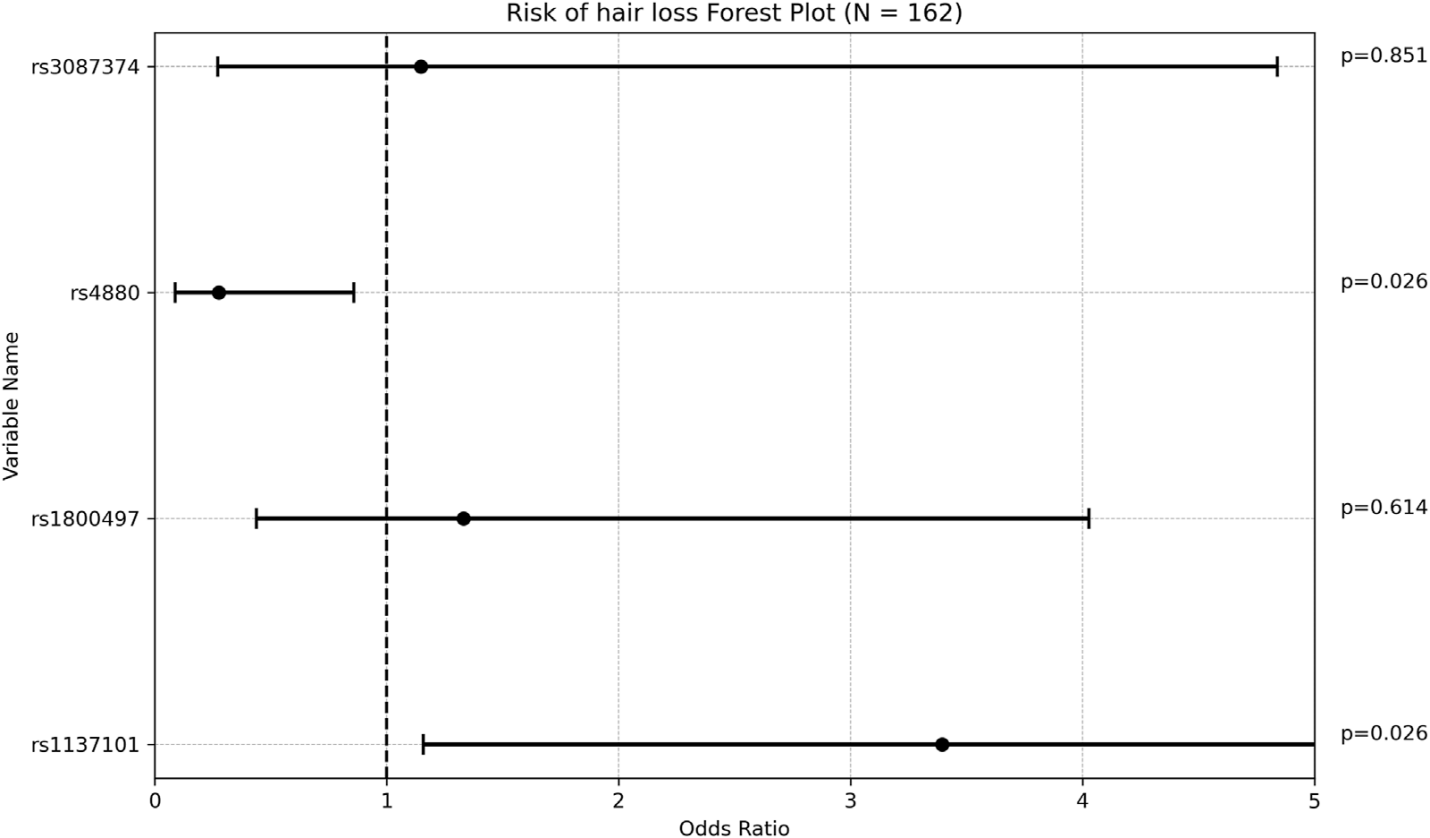
Forest plot representing the multivariate odds ratios (95%CI) for hair loss in each SNP.

Regarding hair loss, patients who experienced this side effect had a significantly lower mean maintenance VPA dose compared to those who did not (13.38 ± 4.00 mg/kg/d vs 17.38 ± 8.04 mg/kg/d, respectively; *p* = 0.001. This finding is however inconclusive due to small percentage of patients who reported hair loss (Supplementary Table S6). A majority of patients (78.95%) who reported hair loss were females, compared to 41.26% in the group who did not report this side effect (*p* = 0.002). This finding is expected as females are more likely to notice and report hair loss. No other potential confounding factor was significantly associated with hair loss. The *rs1137101 (LEPR)* allele was present in 68.42% of patients with hair loss, compared to 40.56% of those without hair loss (*p* = 0.021). After adjusting for gender and VPA maintenance dose, the OR was 3.394 [1.157; 9.956], indicating that patients with this genetic variant had approximately three times the risk of developing hair loss. Conversely, rs4880 *(SOD2)* was found in 73.43% of patients without hair loss compared to 52.63% of those with hair loss (*p* = 0.061). This association became statistically significant when adjusting for gender and VPA maintenance dose, with a multivariate OR of 0.276 [0.089; 0.858], suggesting a protective effect of this SNP against hair loss. None of the remaining SNPs analyzed were significantly associated with hair loss (Figure 3).

### 3.5 Association with VPA concentrations

**FIGURE 4:**
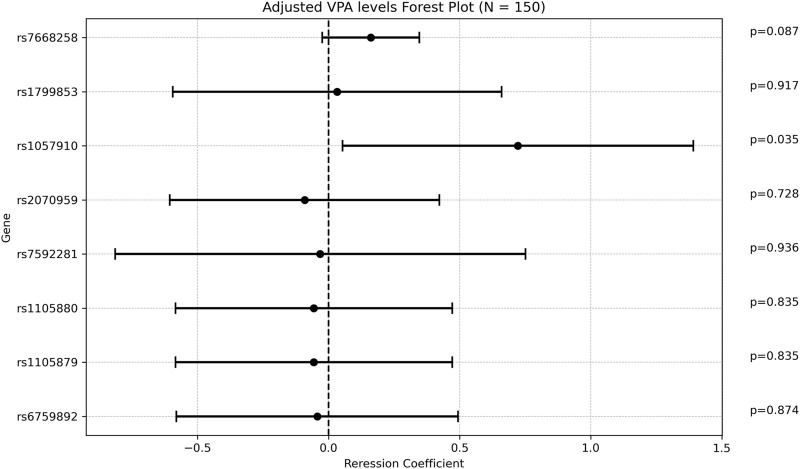
Forest plot representing the multivariate regression coefficients (95%CI) for ADC in each SNP.

There was a weak positive correlation between age at diagnosis and ADC, with a regression coefficient of 0.048 [0.011; 0.084], *p* = 0.0104 (Supplementary Table S7). The *rs1057910* variant (CYP2C9*3) was associated with increased ADC with a mean of 4.9 ± 1.75 [(ug/mL/day)/(mg/kg)] in carriers vs 4.15 ± 1.61 in wildtype individuals (*p* = 0.028). In multivariate analysis, the regression coefficient was 0.722 [0.053; 1.391], *p* = 0.034. Of note that the variant rs7668258 (*UGT2B7*) was significantly associated with ADC only in the univariate analysis. None of the remaining SNPs were found to be significantly associated with ADC (Figure 4).

## 4 Discussion

Our study identified a significant association between the efficacy of VPA and the *rs3892097* variant *(CYP2D6)*. Furthermore, we found that adjusted serum VPA concentrations were impacted by the presence of the *rs1057910* variant (CYP2C9*3). Additionally, certain adverse effects of VPA were found to be influenced by the individual’s genetic profile. Specifically, weight gain was associated with the *rs1137101 (LEPR)*, while hair loss was affected by the rs1137101 *(LEPR)* and rs4480 variants *(SOD2).*

Our findings revealed that *rs3892097* variant in the *CYP2D6* gene was associated with an increased rate of treatment failure. The *CYP2D6* enzyme (debrisoquine hydroxylase) is involved in the metabolism of several antidepressants and neuroleptics (Kirchheiner et al., 2001; Wedlund and de Leon, 2004; Rau et al., 2004). Genetic variations in this enzyme significantly affect drug metabolism rates, leading to the categorization of different metabolizer groups: ultrarapid, extensive, intermediate, and poor metabolizers, which necessitates dosage adjustments (Xie et al., 2001; Shastry, 2005). While data on the interaction between the *rs3892097* variant (*CYP2D6*) and VPA is limited, existing literature supports our findings. One study has previously linked the presence of *rs3892097 (CYP2D6)* to refractory epilepsy in pediatric patients (López-García et al., 2017). Our results are consistent with these observations, and a possible explanation could be the impaired VPA oxidation and high toxic metabolites levels associated with the *rs3892097* variant (Shnayder et al., 2023).

Conversely, the *rs1057910* variant of the *CYP2C9* gene (also known as CYP2C9*3) was associated with significantly higher VPA serum concentrations compared to wild type cases. The *CYP2C9* enzyme participates in the metabolism of different classes of drugs (CYP2C9 cytochrome, 2024) and is the primary enzyme responsible for the hydroxylation and desaturation of VPA (Kiang et al., 2006). Our findings are corroborated by a meta-analysis, which demonstrated that individuals heterozygous for the CYP2C9*3 polymorphism had higher VPA serum concentrations compared to homozygous wild type individuals (Yoon et al., 2020). Additionally, one study highlighted an inverse relationship between VPA serum concentrations and the seizure frequency (Gram et al., 1979).

Hair loss is a common side effect of VPA, with reported incidences ranging from 3.5% to 12% (Kakunje et al., 2018). Proposed mechanisms for VPA-induced hair loss include deficiencies in biotin, vitamin D, and other trace minerals, such as zinc, iron, copper and magnesium (Kakunje et al., 2018; Wolff et al., 2008; Plonka et al., 2005; Kil et al., 2013; Praharaj et al., 2022). Another suggested mechanism is VPA induced telogen effluvium, which may be linked to its aromatase-inhibiting effects, potentially leading to male pattern baldness through subsequent hyperandrogenism (Praharaj et al., 2022). In our study, the *rs1137101* variant *(LEPR)* was associated with a significant increased risk of hair loss, a novel finding that has not been previously reported. Given that the leptin receptor plays an important role in inducing the anagen (growth) phase of the hair follicle (Sumikawa et al., 2014), it is plausible that the *rs1137101* variant *(LEPR)* is associated with a defective leptin receptor. This defect could impair the initiation of the anagen phase, thereby increasing susceptibility to VPA-induced hair loss. Furthermore, our results indicated that patients with the *rs4480* variant of the *SOD2* gene was associated with a lower incidence of hair loss, suggesting a protective effect. The *SOD2* encodes superoxide dismutase 2, a mitochondrial scavenging enzyme that converts superoxide byproducts of oxidative phosphorylation to the less reactive hydrogen peroxide (H2O2) and oxygen (O2) (Superoxide dismutase, 2024b). The *rs4480 (SOD2)* SNP (Val16Ala) yields the Ala variant, which is more efficiently imported into the mitochondrial matrix compared to the Val variant, thereby enhancing mitochondrial SOD2 activity (Sutton et al., 2003). Consistent with this, previous research has linked the Val to Ala polymorphism with a reduced risk of hepatotoxicity compared to the Val/Val genotype (Saruwatari et al., 2012). Our study is the first to relate *rs4480 (SOD2)* to VPA-induced hair loss. There is however circumstantial evidence suggesting that increased oxidative stress contributes to hair follicle damage and hair loss (Trüeb, 2009; Trüeb, 2021). VPA has been shown to increase oxidative stress in a study involving pediatric patients (Verrotti et al., 2008). It is therefore possible that the protective effect of increased *SOD2* activity counteracts the oxidative stress effects induced by VPA, thereby mitigating hair loss. Additionally, VPA-induced deficiencies in trace elements like zinc and copper, which are cofactors for *SOD1* (Superoxide dismutase, 2024a), may exacerbate oxidative stress and hair loss. Therefore, the enhanced *SOD2* activity conferred by the *rs4480* variant *(SOD2)* could help offset these adverse effects.

Our findings further reveal that the presence of *rs1137101* variant *(LEPR)* was associated with an increased risk of weight gain while on VPA monotherapy. This SNP variant is located in the *LEPR* gene which encodes the leptin receptor, a key player in regulating metabolism and energy balance. The leptin receptor is responsible for signaling the brain about the body’s energy stores, influencing appetite and energy expenditure. Variants in the LEPR gene, such as *rs1137101 (LEPR)*, can disrupt leptin signaling, potentially leading to leptin resistance, where the brain becomes less responsive to leptin’s regulatory effects. This disruption can interfere with metabolic pathways involved in glucose homeostasis and lipid metabolism, contributing to weight gain. Mutations in the *LEPR* gene have been associated with obesity and type II diabetes mellitus (Leptin receptor, 2024; Supti et al., 2024; Veerabathiran et al., 2023). Supporting our findings, a positive association between weight gain and the *LEPR rs1137101* SNP was also demonstrated in a study on 212 epilepsy patients receiving VPA (Li et al., 2015).

Our study has several significant limitations. Notably, the relatively small sample size may have contributed to wide confidence intervals in our findings. Additionally, despite being a prospective cohort study, we relied on data extracted from patient medical records, which inherently includes potential biases and inaccuracies. Another limitation is the subjective nature of some reported outcomes, particularly side effects, which are prone to recall bias. Finally, in a population with varied ages and weights like the one in this study, one would ideally use a population pharmacokinetic model to estimate VPA clearance for each individual (Mould and Upton, 2013). Such estimates would be more accurate and allow for more robust correlations with genetic factors. Despite these limitations, our study has notable strengths. It is among the first to explore the genetic variants *rs1057910* (CYP2C9*3)*, rs1137101 (LEPR), rs4480(SOD2)*, and *rs3892097 (CYP2D6)* in the context of VPA treatment effectiveness and side effects. Our use of multivariate analysis adjusted for confounding factors, such as maintenance VPA dose, strengthens the validity of our conclusions.

## 5 Conclusions

In conclusion, our findings underscore the significant role of genetic variations in influencing the efficacy and adverse effects of VPA treatment for epilepsy. We identified a strong association between the rs3892097 *(CYP2D6)* variant and treatment failure, aligning with previous studies that suggest its role in drug metabolism. Additionally, we showed that carriers of the *rs1057910* variants had higher adjusted VPA serum concentrations. Furthermore, the observed associations between *rs1137101 (LEPR)* and *rs4480* variants *(SOD2)* with weight gain and hair loss further highlight the importance of personalized medicine in managing epilepsy. These insights into genetic influences on VPA treatment can guide more tailored therapeutic approaches, improving patient outcomes and minimizing adverse effects. Our study emphasizes the need for further research to elucidate the mechanistic pathways of these genetic variants and validate our findings in larger, prospective cohorts.

## Data Availability

The datasets presented in this study can be found in online repositories. The names of the repository/repositories and accession number(s) can be found in the article/Supplementary Material.
