# Supplementary material for: The role of candidate pharmacogenetic variants in determining valproic acid efficacy, toxicity and concentrations in patients with epilepsy
Source: Front Pharmacol. 2024 Oct 30;15:1483723. doi: 10.3389/fphar.2024.1483723 (PMC11558073; doi:10.3389/fphar.2024.1483723)
Supplement: Supplementary file 1 [file Table1.docx]

**Supplementary table 1.** Genes variants characteristics

| **Variant (rs)** | **Gene** | **Phenotype Categories** | **Association analysis** | **Classification (as per dbSNP)** | **Reference allele** | **Alternative allele** | **Alternative allele frequency (%)**  **(in sample, N = 166)** | **Alternative Allele frequency (%) (GNOMAD Genome, all populations)** | **Alternative Allele frequency (%) (GNOMAD Genome, NFE)** | **Sequencing status** | **HWE P-values** |
| --- | --- | --- | --- | --- | --- | --- | --- | --- | --- | --- | --- |
| *rs1000940* | *RABEP1* | Other | - | Intron | A | C,G,T | N/A | 31.6 (G) | 30.35 (G) | **Not sequenced** | N/A |
| *rs1019385* | *GRIN2B* | Dosage | - | Splice region and intron variant | C | A | 14.525 | 36.85 | 45.59 | Sequenced | **<0.0001** |
| *rs10445704* | *UGT1A6* | Dosage | - | 5 prime Upstream transcript variant | G | A | 1.117 | 39.33 | 40.11 | Sequenced | **<0.0001** |
| *rs1057910* | *CYP2C9* | Dosage, Metabolism/PK | VPA adjusted level | Missense | A | C,G | 9.777 (C) | 4.95 (C)  0.00 (G) | 6.62 (C)  0.00 (G) | Sequenced | 0.275 |
| *rs1065852* | *CYP2D6* | Efficacy | - | Missense | G | A,C | 16.76 (A) | 19.06 (A) | 22.25 (A) | Sequenced | **0.008** |
| *rs1105879* | *UGT1A6* | Dosage | VPA adjusted level | Missense | A | C | 37.989 | 33.65 | 34.8 | Sequenced | 0.792 |
| *rs1105880* | *UGT1A6* | Dosage | VPA adjusted level | Synonymous variant | A | G | 37.989 | 35.48 | 34.82 | Sequenced | 0.792 |
| *rs1137101* | *LEPR* | Toxicity | Toxicity | Missense | A | G,T | 24.581 (G) | 50.34 (G) | 45.26 (G) | Sequenced | 0.256 |
| *rs12233719* | *UGT2B7* | Metabolism/PK | - | Missense | G | A,C,T | N/A | 0.67 (T) | 0.01 (T) | **No mutation found** | N/A |
| *rs1799853* | *CYP2C9* | Dosage, Metabolism/PK | VPA adjusted level | Missense | C | T | 11.732 | 8.86 | 12.76 | Sequenced | 0.267 |
| *rs1800012* | *COL1A1* | Toxicity | - | Intron | C | A | N/A | 13.70 | 18.60 | **Not Sequenced** | N/A |
| *rs1800497* | *ANKK1* | Toxicity | Toxicity | Missense | G | A | 15.363 | 25.78 | 19.17 | Sequenced | 0.897 |
| *rs2070959* | *UGT1A6, UGT1A10; UGT1A7; UGT1A8; UGT1A9* | Dosage | VPA adjusted level | Missence | A | G | 33.24 | 29.97 | 32.66 | Sequenced | 0.349 |
| *rs2269577* | *XBP1* | Efficacy | Effectiveness | Intron variant and non-coding transcript variant | G | A,C | 35.475 (C) | 39.71 (C) | 30.72 (C) | Sequenced | 0.863 |
| *rs2279020* | *GABRA1* | Efficacy | Effectiveness | Intron | G | A | 55.028 | 37.32 | 64.99 | Sequenced | 0.251 |
| *rs2307441* | *POLG* | Toxicity | - | Missense | T | C | 1.676 | 2.69 | 4.16 | Sequenced | **<0.0001** |
| *rs2606345* | *CYP1A1* | Efficacy | - | Intron | C | A | N/A | 45.70 | 32.65 | **Not Sequenced** | N/A |
| *rs2687116* | *CYP3A4* | Efficacy | - | Intron | C | A,G,T | 96.089 (A) | 79.91 (A) | 96.43 (A) | **No homozygous wildtypes in our cohort** | 0.586 |
| *rs2740574* | *CYP3A4* | Efficacy | - | 2KB upstream variant | C | A,G,T | N/A | 79.10 (T) | 96.49 (T) | **Mutation not called (very low coverage)** | N/A |
| *rs2769605* | *none* | Efficacy | - | Intergenic variant | C | T | N/A | 43.92 | 56.48 | **Mutation not called (very low coverage)** | N/A |
| *rs28898617* | *UGT1A10; UGT1A3; UGT1A4; UGT1A5; UGT1A6; UGT1A7; UGT1A8; UGT1A9* | Metabolism/PK | - | Missense | A | G | N/A | 0.11 | 0 | **No mutation found** | N/A |
| *rs3087374* | *POLG* | Toxicity | Toxicity | Missense | C | A | 7.263 | 6.18 | 8.24 | Sequenced | 0.241 |
| *rs3816877* | *APEH* | Metabolism/PK | - | Missense | C | G,T | N/A | 0.15 (T) | 0.01 (T) | **Mutation not called** | N/A |
| *rs3888190* | *SH2B1* | Toxicity | - | Noncoding transcript exon variant | C | A,T | N/A | 34.31 (A) | 39.38 (A) | **Not sequenced** | N/A |
| *rs3892097* | *CYP2D6* | Efficacy | Effectiveness | Splice Acceptor | C | T | 10.615 | 14.27 | 19.69 | Sequenced | 0.118 |
| *rs4880* | *SOD2* | Toxicity | Toxicity | Missense | A | G | 45.531 | 47.33 | 49.67 | Sequenced | 0.739 |
| *rs6759892* | *UGT1A6, UGT1A10; UGT1A7; UGT1A8; UGT1A9* | Dosage | VPA adjusted level | Missense | T | G | 41.62 | 39.44 | 40.64 | Sequenced | 0.357 |
| *rs7438284* | *UGT2B7* | Efficacy | Effectiveness | Synonymous variant | A | T | 47.207 | 57.55 | 46.76 | Sequenced | 0.067 |
| *rs7592281* | *UGT1A6* | Dosage | VPA adjusted level | Intron | G | A,T | 6.425 (T) | 0.00 (A)  6.39 (T) | 0.01 (A)  2.76 (T) | Sequenced | 0.358 |
| *rs7668258* | *UGT2B7* | Metabolism/PK | VPA adjusted level | Intron | T | A,C,G | 47.207 (C) | 57.68 (C) | 46.86 (C) | Sequenced | 0.067 |
| *rs9332120* | *CYP2C9* | Efficacy | Effectiveness | Intron | T | C | 18.436 | 19.09 | 21.71 | Sequenced | 0.3 |

**Supplementary table 2**. Association of genes variants with treatment efficacy at 12 months with univariate and multivariate logistic regression

| **Variable name** | **Univariate Logistic Regression (N = 166)** | | **Multivariate Logistic Regression (N = 166)** | |
| --- | --- | --- | --- | --- |
|  | **Odds Ratio [95% CI]** | **P-value** | **Odds Ratio [95% CI]** | **P-value** |
| rs2279020 (Wildtype) | ref | ref | ref | ref |
| rs2279020 (Allele carrier + Homozygous mutant) | 1.564 [0.738; 3.314] | 0.243 | 1.587 [0.712; 3.538] | 0.259 |
| rs9332120 (Wildtype) | ref | ref | ref | ref |
| rs9332120 (Allele carrier + Homozygous mutant) | 0.591 [0.307; 1.139] | 0.116 | 0.686 [0.342; 1.375] | 0.288 |
| rs3892097 (Wildtype) | ref | ref | ref | ref |
| rs3892097 (Allele carrier + Homozygous mutant) | 0.453 [0.206; 1.0] | **0.05** | 0.389 [0.169; 0.894] | **0.026** |
| rs7438284 (Wildtype) | ref | ref | ref | ref |
| rs7438284 (Allele carrier + Homozygous mutant) | 0.799 [0.404; 1.58] | 0.519 | 0.776 [0.38; 1.584] | 0.486 |
| rs2269577 (Wildtype) | ref | ref | ref | ref |
| rs2269577 (Allele carrier + Homozygous mutant) | 0.727 [0.379; 1.392] | 0.336 | 0.783 [0.393; 1.562] | 0.487 |

Multivariate regression took into consideration demographics that were statistically significant at the univariate level.

**Supplementary table 3.** Association of demographics and genes variants and nausea and or vomiting with univariate and multivariate logistic regression

| **Variable name** | **All patients (N=162)** | **No nausea and/or vomiting (N=154)** | **Nausea and/or vomiting (N=8)** | **P-value** | **Univariate Logistic Regression** | | **Multivariate Logistic Regression** | |
| --- | --- | --- | --- | --- | --- | --- | --- | --- |
|  |  |  |  |  | **Odds Ratio [95% CI]** | **P-value** | **Odds Ratio [95% CI]** | **P-value** |
| Age at visit 1 (years) | 12.81 ± 7.26 | 12.56 ± 7.11 | 17.62 ± 8.93 | 0.061 | - | - | - | - |
| Maintenance dose per kg per day (mg/kg/d) | 16.91 ± 7.77 | 16.65 ± 7.52 | 22.03 ± 11.00 | 0.101 | - | - | - | - |
| Follow-up period (years) | 6.59 ± 2.61 | 6.49 ± 2.63 | 8.33 ± 0.91 | 0.081 | - | - | - | - |
| Gender (Female) | 74 (45.68) | 66 (42.86) | 8 (100.00) | **0.002** | - | - | - | - |
| Gender (Male) | 88 (54.32) | 88 (57.14) | 0 (0.00) | - | - | - | - | - |
| rs1137101 (Wildtype) | 91 (56.17) | 87 (56.49) | 4 (50.00) | 0.718 | ref | ref | ref | ref |
| rs1137101 (Allele carrier + Homozygous mutant) | 71 (43.83) | 67 (43.51) | 4 (50.00) | - | 1.299 [0.313; 5.383] | 0.719 | 1.2 [0.276; 5.209] | 0.808 |
| rs1800497 (Wildtype) | 117 (72.22) | 110 (71.43) | 7 (87.50) | 0.322 | ref | ref | ref | ref |
| rs1800497 (Allele carrier + Homozygous mutant) | 45 (27.78) | 44 (28.57) | 1 (12.50) | - | 0.357 [0.043; 2.988] | 0.342 | 0.353 [0.041; 3.07] | 0.346 |
| rs4880 (Wildtype) | 47 (29.01) | 45 (29.22) | 2 (25.00) | 0.798 | ref | ref | ref | ref |
| rs4880 (Allele carrier + Homozygous mutant) | 115 (70.99) | 109 (70.78) | 6 (75.00) | - | 1.239 [0.241; 6.369] | 0.798 | 1.304 [0.242; 7.028] | 0.757 |
| rs3087374 (Wildtype) | 139 (85.80) | 133 (86.36) | 6 (75.00) | 0.369 | ref | ref | ref | ref |
| rs3087374 (Allele carrier + Homozygous mutant) | 23 (14.20) | 21 (13.64) | 2 (25.00) | - | 2.111 [0.399; 11.161] | 0.379 | 1.5 [0.269; 8.362] | 0.644 |

Mann-whitney U test was used for continuous variables and Chi-square for categorical ones. Continuous variables were reported as mean ± SD. Categorical variables were reported as N (%)

Multivariate regression took into consideration demographics that were statistically significant at the univariate level.

**Supplementary table** **4**. Association of demographics and genes variants and tremor with univariate logistic regression

| **Variable name** | **All patients (N=163)** | **No Tremor (N=143)** | **Tremor (N=20)** | **P-value** | **Univariate Logistic Regression** | | **Multivariate Logistic Regression** | |
| --- | --- | --- | --- | --- | --- | --- | --- | --- |
|  |  |  |  |  | **Odds Ratio [95% CI]** | **P-value [95% CI]** | **Odds Ratio [95% CI]** | **P-value [95% CI]** |
| Age at visit 1 (years) | 12.77 ± 7.25 | 12.37 ± 7.20 | 15.68 ± 7.17 | **0.046** | - | - | - | - |
| Maintenance dose per kg per day (mg/kg/d) | 17.01 ± 7.84 | 17.13 ± 8.03 | 16.15 ± 6.47 | 0.576 | - | - | - | - |
| Follow-up period (years) | 6.61 ± 2.61 | 6.54 ± 2.68 | 7.13 ± 2.10 | 0.338 | - | - | - | - |
| Gender (Female) | 75 (46.01) | 62 (43.36) | 13 (65.00) | 0.069 | - | - | - | - |
| Gender (Male) | 88 (53.99) | 81 (56.64) | 7 (35.00) | - | - | - | - | - |
| rs1137101 (Wildtype) | 91 (55.83) | 81 (56.64) | 10 (50.00) | 0.575 | ref | ref | ref | ref |
| rs1137101 (Allele carrier + Homozygous mutant) | 72 (44.17) | 62 (43.36) | 10 (50.00) | - | 1.306 [0.512; 3.334] | 0.576 | 1.277 [0.495; 3.297] | 0.613 |
| rs1800497 (Wildtype) | 118 (72.39) | 101 (70.63) | 17 (85.00) | 0.178 | ref | ref | ref | ref |
| rs1800497 (Allele carrier + Homozygous mutant) | 45 (27.61) | 42 (29.37) | 3 (15.00) | - | 0.424 [0.118; 1.525] | 0.189 | 0.401 [0.109; 1.472] | 0.169 |
| rs4880 (Wildtype) | 47 (28.83) | 40 (27.97) | 7 (35.00) | 0.516 | ref | ref | ref | ref |
| rs4880 (Allele carrier + Homozygous mutant) | 116 (71.17) | 103 (72.03) | 13 (65.00) | - | 0.721 [0.268; 1.939] | 0.517 | 0.658 [0.240; 1.803] | 0.416 |
| rs3087374 (Wildtype) | 140 (85.89) | 122 (85.31) | 18 (90.00) | 0.573 | ref | ref | ref | ref |
| rs3087374 (Allele carrier + Homozygous mutant) | 23 (14.11) | 21 (14.69) | 2 (10.00) | - | 0.646 [0.139; 2.989] | 0.576 | 0.572 [0.121; 2.704] | 0.481 |

Mann-whitney U test was used for continuous variables and Chi-square for categorical ones. Continuous variables were reported as mean ± SD. Categorical variables were reported as N (%)

**Supplementary table 5**. Association of demographics and genes variants and weight gain with univariate and multivariate linear regression

|  | **Univariate Linear Regression (N = 163)** | | | | **Multivariate Linear Regression (N = 163)** | | | | **Weight gain (N = 163)** | | |
| --- | --- | --- | --- | --- | --- | --- | --- | --- | --- | --- | --- |
| **Variable name** | **Coefficient** | **lower CI** | **upper CI** | **P-value** | **Coefficient** | **lower CI** | **upper CI** | **P-value** | **Wild type** | **Variant allele carriers** | **P-Value** |
| Age at visit 1 (years) | -0.192 | -0.403 | 0.020 | 0.076 | - | - | - | - | - | - | - |
| Gender | 1.455 | -1.582 | 4.493 | 0.345 | - | - | - | - | - | - | - |
| Maintenance dose per kg per day (mg/kg/d) | -0.401 | -0.587 | -0.216 | **<0.0001** | - | - | - | - | - | - | - |
| Follow-up period (years) | 0.612 | 0.032 | 1.192 | **0.039** | - | - | - | - | - | - | - |
| rs1137101 | 1.672 | -1.369 | 4.713 | 0.279 | 3.430 | 0.674 | 6.186 | **0.015** | 8.44 ± 9.03 | 10.11 ± 10.63 | 0.373 |
| rs1800497 | -0.623 | -4.020 | 2.775 | 0.718 | -1.239 | -4.306 | 1.827 | 0.426 | 9.37 ± 10.37 | 8.75 ± 8.16 | 0.851 |
| rs4880 | -0.210 | -3.524 | 3.103 | 0.900 | -0.498 | -3.522 | 2.525 | 0.745 | 9.35 ± 9.77 | 9.14 ± 9.85 | 0.922 |
| rs3087374 | 0.529 | -3.918 | 4.975 | 0.815 | 2.224 | -1.873 | 6.320 | 0.285 | 9.13 ± 9.75 | 9.66 ± 10.3 | 0.961 |

Continuous variables were reported as mean ± SD and the Mann-Whitney U test was used for the gene variants

Multivariate regression took into consideration demographics that were statistically significant at the univariate level in addition to age a visit 1 and follow-up period to account for children growth

**Supplementary table 6**: Association of demographics and genes variants and hair loss with univariate and multivariate logistic regression

| **Variable name** | **All patients (N=162)** | **No Hair loss (N=143)** | **Hair loss (N=19)** | **P-value** | **Univariate Logistic Regression** | | **Multivariate Logistic Regression** | |
| --- | --- | --- | --- | --- | --- | --- | --- | --- |
|  |  |  |  |  | **Odds Ratio [95% CI]** | **P-value** | **Odds Ratio [95% CI]** | **P-value** |
| Age at visit 1 (years) | 12.81 ± 7.26 | 12.50 ± 7.20 | 15.11 ± 7.51 | 0.165 | - | - | - | - |
| Maintenance dose per kg per day (mg/kg/d) | 16.91 ± 7.77 | 17.38 ± 8.04 | 13.38 ± 4.00 | **0.017** | - | - | - | - |
| Follow-up period (years) | 6.59 ± 2.61 | 6.50 ± 2.60 | 7.26 ± 2.59 | 0.211 | - | - | - | - |
| Gender (Female) | 74 (45.68) | 59 (41.26) | 15 (78.95) | **0.002** | - | - | - | - |
| Gender (Male) | 88 (54.32) | 84 (58.74) | 4 (21.05) | - | - | - | - | - |
| rs1137101 (Wildtype) | 91 (56.17) | 85 (59.44) | 6 (31.58) | 0.021 | ref | ref | ref | ref |
| rs1137101 (Allele carrier + Homozygous mutant) | 71 (43.83) | 58 (40.56) | 13 (68.42) | - | 3.175 [1.141; 8.835] | **0.027** | 3.394 [1.157; 9.956] | **0.026** |
| rs1800497 (Wildtype) | 117 (72.22) | 105 (73.43) | 12 (63.16) | 0.348 | ref | ref | ref | ref |
| rs1800497 (Allele carrier + Homozygous mutant) | 45 (27.78) | 38 (26.57) | 7 (36.84) | - | 1.612 [0.591; 4.396] | 0.351 | 1.33 [0.439; 4.028] | 0.614 |
| rs4880 (Wildtype) | 47 (29.01) | 38 (26.57) | 9 (47.37) | 0.061 | ref | ref | ref | ref |
| rs4880 (Allele carrier + Homozygous mutant) | 115 (70.99) | 105 (73.43) | 10 (52.63) | - | 0.402 [0.152; 1.065] | 0.067 | 0.276 [0.089; 0.858] | **0.026** |
| rs3087374 (Wildtype) | 139 (85.80) | 123 (86.01) | 16 (84.21) | 0.832 | ref | ref | ref | ref |
| rs3087374 (Allele carrier + Homozygous mutant) | 23 (14.20) | 20 (13.99) | 3 (15.79) | - | 1.153 [0.308; 4.319] | 0.833 | 1.147 [0.272; 4.84] | 0.851 |

Mann-whitney U test was used for continuous variables and Chi-square for categorical ones. Continuous variables were reported as mean ± SD. Categorical variables were reported as N (%)

Multivariate regression took into consideration demographics that were statistically significant at the univariate level.

**Supplementary table 7**. Association of demographics and genes variants and average of Adjusted VPA concentrations with univariate and multivariate linear regression

|  | **Univariate Linear Regression (N = 150)** | | | | **Multivariate Linear Regression (N = 150)** | | | | **Average of Adjusted VPA concentrations ((ug/mL/day)/(mg/kg)) (N = 150)** | | |
| --- | --- | --- | --- | --- | --- | --- | --- | --- | --- | --- | --- |
| **Variable name** | **Coefficient** | **lower CI** | **upper CI** | **P-value** | **Coefficient** | **lower CI** | **upper CI** | **P-value** | **Wild type** | **Variant allele carriers** | **P-Value** |
| Age at visit 1 (years) | 0.048 | 0.011 | 0.084 | 0.0104 | - | - | - | - | - | - | - |
| Gender | -0.441 | -0.974 | 0.093 | 0.1049 | - | - | - | - | - | - | - |
| Follow-up period (years) | 0.021 | -0.082 | 0.123 | 0.6879 | - | - | - | - | - | - | - |
| rs6759892 | -0.010 | -0.574 | 0.555 | 0.9724 | -0.043 | -0.580 | 0.494 | 0.8738 | 4.29 ± 1.7 | 4.28 ± 1.65 | 0.778 |
| rs1105879 | -0.029 | -0.585 | 0.526 | 0.9172 | -0.056 | -0.584 | 0.472 | 0.8352 | 4.31 ± 1.64 | 4.28 ± 1.68 | 0.896 |
| rs1105880 | -0.029 | -0.585 | 0.526 | 0.9172 | -0.056 | -0.584 | 0.472 | 0.8352 | 4.31 ± 1.64 | 4.28 ± 1.68 | 0.896 |
| rs7592281 | 0.062 | -0.746 | 0.869 | 0.8805 | -0.032 | -0.814 | 0.751 | 0.9363 | 4.28 ± 1.63 | 4.34 ± 1.88 | 0.932 |
| rs2070959 | -0.149 | -0.692 | 0.394 | 0.5883 | -0.091 | -0.605 | 0.423 | 0.7276 | 4.37 ± 1.68 | 4.22 ± 1.65 | 0.728 |
| rs1057910 | 0.747 | 0.058 | 1.435 | **0.0337** | 0.722 | 0.053 | 1.391 | **0.0345** | 4.15 ± 1.61 | 4.9 ± 1.75 | **0.028** |
| rs1799853 | -0.178 | -0.833 | 0.478 | 0.593 | 0.033 | -0.594 | 0.660 | 0.9174 | 4.33 ± 1.65 | 4.15 ± 1.7 | 0.51 |
| rs7668258 | 0.608 | 0.030 | 1.185 | 0.0395 | 0.161 | -0.024 | 0.346 | 0.0871 | 3.86 ± 1.61 | 4.47 ± 1.65 | 0.033 |

Continuous variables were reported as mean ± SD and the Mann-Whitney U test was used for the gene variants

Multivariate regression took into consideration demographics that were statistically significant at the univariate level
